# Bibliographical

**Published:** 1883-01

**Authors:** 


### Bibliographical.

[Elements of DENTAL MATERIA MEDICA and THERAPEUTICS with PHARMACOPOEIA. Third Edition, by JAMES STOCKEN, L.D.S., Eng., assisted by THOMAS GADDES, L.D.S., Eng. and Edn.]

With the former editions of this work the dental profession is, for the most part, familiar. The work has been highly appreciated and justly so.

The work has been thoroughly revised, and in many instances important additions have been made, and several new preparations have been inserted. An "Index of Diseases" has been compiled, the advantages of which will be very manifest. The aim has been to bring this edition to the present standard of knowledge, and in keeping with the rapid progress of the dental profession.

This is the only work of any pretensions prepared especially for the dentist's use, and it should be in the library of every dental practitioner. There are very few, if any, who can hold and retain in memory, ready for use at any moment, all facts and knowledge pertaining to the practice of medicine or any of its departments or specialties, hence the importance of helps and reference books such as this, which should always be at hand, and frequently consulted. The work is published by P. Blakiston & Son, and may be obtained through any bookseller.

